# Nanopore Detector based analysis of single-molecule conformational kinetics and binding interactions

**DOI:** 10.1186/1471-2105-7-S2-S21

**Published:** 2006-09-26

**Authors:** Stephen Winters-Hilt

**Affiliations:** 1 Department of Computer Science, University of New Orleans, New Orleans, LA 70148, USA; 2 The Research Institute for Children, 200 Henry Clay Ave., New Orleans, LA 70118, USA

## Abstract

**Background:**

A Nanopore Detector provides a means to transduce single molecule events into observable channel current changes. Nanopore-based detection can report directly, or indirectly, on single molecule kinetics. The nanopore-based detector can *directly *measure molecular characteristics in terms of the blockade properties of individual molecules – this is possible due to the kinetic information that is embedded in the blockade measurements, where the adsorption-desorption history of the molecule to the surrounding channel, and the configurational changes in the molecule itself, imprint on the ionic flow through the channel. This rich source of information offers prospects for DNA sequencing and single nucleotide polymorphism (SNP) analysis. A nanopore-based detector can also measure molecular characteristics *indirectly*, by using a reporter molecule that binds to certain molecules, with subsequent distinctive blockade by the bound-molecule complex.

**Results:**

It is hypothesized that reaction histories of *individual *molecules can be observed on model DNA/DNA, DNA/Protein, and Protein/Protein systems. Preliminary results are all consistent with this hypothesis. Nanopore detection capabilities are also described for highly discriminatory biosensing, binding strength characterization, and rapid immunological screening.

**Conclusion:**

In essence, the heart of chemistry is now accessible to a new, single-molecule, observation method that can track both external molecular binding states, and internal conformation states.

## Background

### A New Method for Single Molecule Detection and Characterization

Angstrom precision structures for numerous DNA, RNA, and protein molecules have been revealed by X-ray diffraction analysis and NMR spectroscopy. These approaches rely upon average properties of very large numbers of molecules and are often biased towards crystallization and NMR conformer structures different from those present in solution under any conditions, physiological conditions in particular. With the introduction of atomic force microscopy and laser tweezers in the early 1990's three direct measures of the force have been performed at the single molecule level: (1) the force required to break A•T or G•C base pairs [1-3], (2) the force required to extend single or double stranded DNA through distinct structural conformations, e.g., B form to S form DNA [4,5], etc., and (3) the forces exerted by polymerases working on polynucleotides [6]. Single molecule analytical techniques, however, have yet to offer a means to directly observe *single *molecule binding *histories *for molecules in solution. Brief snapshots of binding events, often with significant time-averaging of events, are possible with molecular beacon approaches, but these approaches come no where near matching the potential of a nanopore detector to observe single molecules for extensive periods, unmodified by chromophore attachment, etc. The nanopore detector also presents the possibility of observing conformational change *within *a molecule (see [26] for latest results), something not easily addressed by other methods.

Channel current based nanopore cheminformatics provides an incredibly versatile method for transducing single molecule events into discernable channel current blockade levels (states). Single biomolecules, and the ends of biopolymers such as DNA, have been examined in solution with nanometer-scale precision [7-12]. In early studies [12], it was found that complete base-pair dissociations of dsDNA to ssDNA, "melting", could be observed for sufficiently short DNA hairpins. In later work [9,11], the nanopore detector attained Angstrom resolution and was used to "read" the ends of dsDNA molecules, and was operated as a chemical biosensor. In recent work [7,8,10], the nanopore detector is being used to observe the conformational kinetics at the termini of single DNA molecules. And in the most recent work, reported here, information on single-molecule binding and conformational kinetics is obtained by observation of single-molecule channel blockade currents. The DNA-DNA, DNA-protein, and protein-protein binding experiments that are described are novel in that they make critical use of indirect sensing (described below), where one of the molecules in the binding experiment is either a natural channel blockade "toggler", i.e., not residing in a non-informative "stuck" state, or makes use of an attached auxiliary molecule that provides a multi-level blockade when it is captured in the channel.

### The Coulter Counter

The notion of using channels as detection devices dates back to the Coulter counter [13], where pulses in channel flow were measured in order to count bacterial cells. Cell transport through the Coulter counter is driven by hydrostatic pressure – and interactions between the cells and the walls of the channel are ignored. Since its original formulation, channel sizes have reduced from millimeter scale to nanometer scale, and the detection mechanism has shifted from measurements of hydrostatically driven fluid flow to measurements of electrophoretically driven ion flow. Analytes observed via channel measurements are likewise reduced in scale, and are now at the scale of single biomolecules such as DNA and polypeptides [7-12,14-19]. To a limited extent, some intramolecular, Angstrom-level, features are beginning to be resolved as well [7-11].

For nanoscopic channels, interactions between channel wall and translocating biomolecules cannot, usually, be ignored. On the one hand this complicates analysis of channel blockade signals, on the other hand, tell-tale on-off kinetics are revealed for binding between analyte and channel, and this is what has allowed the probing of intramolecular structure on single DNA molecules [7-11].

### Coulter Data – Blockades typically static

Biophysicists and medical researchers have performed measurements of ion flow through single nanopores since the 1970's. It was they who first designed the sensitive patch clamp amplifiers needed for the picoampere ionic current measurements [20,21]. Typically, these single channel techniques have been applied to low conductance, ion selective, channels, such as gated potassium channels, but they have also been applied to larger channels involved in metabolite and macromolecule transport. The use of large biological pores as polymer sensors is a relatively new possibility that dates from the pioneering experiments of Bezrukov et al., 1994 [19]. Their work proved that resistive pulse measurements, *familiar from cell counting with the Coulter counter*[13], could be reduced to the molecular scale and applied to polymers in solution. A seminal paper, by Kasianowicz et al., 1996 [14], then showed that *individual *DNA and RNA polymers could be detected via their translocation blockade of a nanoscale pore formed by α-hemolysin toxin. In such prior nanopore detection work, the data analysis problems were of a familiar "Coulter event" form – where the event was associated with a current blockade at a certain, fixed, level. In the more informative setting, that is possible with nanometer scale channels (due to non-negligible interaction between analyte and channel), the blockading molecule will not provide a *single*, fixed, current reduction in the channel, but will modulate the ion flow through the channel by imprinting its binding and conformational kinetics on the confined channel flow environment.

### The highly stable, nanometer-scale, α-hemolysin protein channel

**Figure 1:**
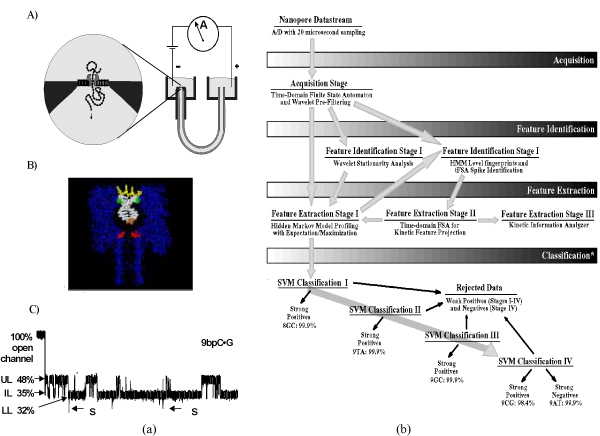
**a**. (A) shows a nanopore device based on the α-hemolysin channel (from [8]). It has been used for analysis of single DNA molecules, such as ssDNA, shown, and dsDNA, a nine base-pair DNA hairpin is shown in (B) superimposed on the channel geometry. The channel current blockade trace for the nine base-pair DNA hairpin blockade from (B) is shown in (C). **b**. The signal processing architecture that was used to classify DNA hairpins with this approach: Signal acquisition was performed using a time-domain, thresholding, Finite State Automaton, followed by adaptive pre-filtering using a wavelet-domain Finite State Automaton. Hidden Markov Model processing with Expectation-Maximization was used for feature extraction on acquired channel blockades. Classification was then done by Support Vector Machine on five DNA molecules: four DNA hairpin molecules with nine base-pair stem lengths that only differed in their blunt-ended DNA termini, and an eight base-pair DNA hairpin. The accuracy shown is obtained upon completing the 15^th ^single molecule sampling/classification (in approx. 6 seconds), where SVM-based rejection on noisy signals was employed.

The α-hemolysin channel is a protein heptamer, formed by seven identical 33 kD protein molecules secreted by *Staphylococcus aureus*. The total channel length is 10 nm and is comprised of a 5 nm *trans*-membrane domain and a 5 nm vestibule that protrudes into the aqueous *cis *compartment [22]. The narrowest segment of the pore is a 1.5 nm-diameter aperture [22], see Figure 1a for crystallographic image. By comparison, a single strand of DNA is about 1.3 nm in diameter. Given that water molecules are 0.15 nm in diameter, this means that one hydration layer separates ssDNA from the amino acids in the limiting aperture. This places the charged phosphodiester backbone, hydrogen bond donors and acceptors, and apolar rings of the DNA bases within one Debye length (3 Å in 1 M KCl) of the pore wall (see Figure 1a). *Not surprisingly, DNA and RNA interaction with the α-hemolysin channel during translocation is non-negligible (but not too strong either, i.e., it is not such that the molecule "gets stuck" – a complication with solid state nanopores). Although dsDNA is too large to translocate, about ten base-pairs at one end can still be drawn into the large cis-side vestibule. This actually permits the most sensitive experiments to date, as the ends of "captured" dsDNA molecules can be observed for extensive periods of time to resolve features *[7-11]. For ssDNA translocation under normal operating conditions [12,14-19], approximately one nucleotide passes the limiting aperture of the channel every microsecond, and a vigorous effort is underway to find ways to slow down and control this translocation process ([17], among others).

### The channel current cheminformatics architecture

Figure 1b shows the prototype signal processing architecture developed in [8]. The processing is designed to rapidly extract useful information from noisy blockade signals using feature extraction protocols, wavelet analysis, Hidden Markov Models (HMMs) and Support Vector Machines (SVMs). For blockade signal acquisition and simple, time-domain, feature-extraction, a Finite State Automaton (FSA) approach is used [23] that is based on tuning a variety of threshold parameters. A generic HMM can be used to characterize current blockades by identifying a sequence of sub-blockades as a sequence of state emissions [44-46]. The parameters of the generic-HMM can then be estimated using a method called Expectation/Maximization, or 'EM" [47], to effect de-noising. The HMM method with EM, denoted HMM/EM, is used in what follows.

**Figure 2:**
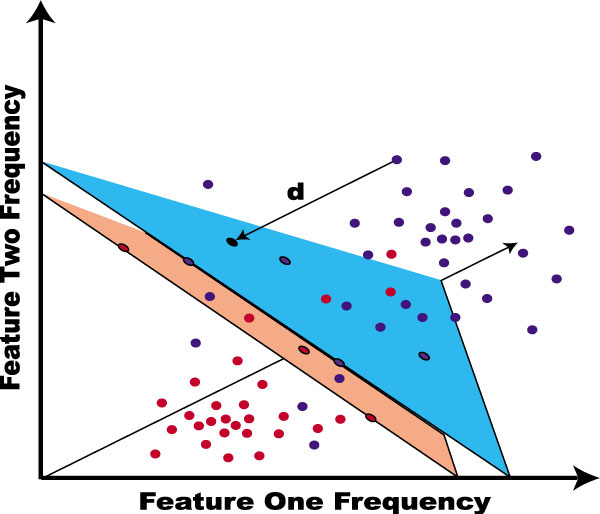
A sketch of the hyperplane separability heuristic for SVM binary classification (from [8]). An SVM is trained to find an optimal hyperplane that separates positive and negative instances, while also constrained by structural risk minimization (SRM) criteria, which here manifests as the hyperplane having a thickness, or "margin," that is made as large as possible in seeking a separating hyperplane. A benefit of using SRM is much less complication due to overfitting (a problem with Neural Network discrimination approaches).

Classification of feature vectors obtained by the HMM for each individual blockade event is then done using SVMs, an approach which automatically provides a confidence measure on each classification. SVMs are fast, easily trained, discriminators [24,25], for which strong discrimination is possible without the over-fitting complications common to neural net discriminators [24]. SVMs strongly draw upon variational methods in their construction and are designed to yield the best estimate of the optimal separating hyperplane (for classifier, see Figure 2) with confidence parameter information included (via hyperplane with margin optimization used in structural risk minimization). The SVM approach also encapsulates a significant amount of model fitting and discriminatory information in the choice of kernel in the SVM, and a number of novel kernels have been developed. In Winters-Hilt et al., 2003 [8], novel, information-theoretic, kernels were introduced for notably better performance over standard kernels – with discrete probability distributions as part of feature vector data. The SVM discriminators are trained by solving their KKT relations using the Sequential Minimal Optimization (SMO) procedure [48]. A chunking [49,50] variant of SMO also is employed to manage the large training task at each SVM node. The multi-class SVM training generally involves thousands of blockade signatures for each signal class.

Different tools are employed at each stage of the signal analysis (as shown in Figure 1b) in order to realize the most robust (and noise resistant) tools for knowledge discovery, information extraction, and classification. Statistical methods for signal rejection using SVMs are also be employed in order to reject extremely noisy signals (see Figure 4 and Figure 5 for previous, five-class, DNA molecule analysis). Since the automated signal processing is based on a variety of machine-learning methods, it is highly adaptable to any type of channel blockade signal. This enables a new type of informatics (cheminformatics) based on channel current measurements, regardless of whether those measurements derive from biologically based or a semiconductor based channels. Further details on the HMM and SVM methods and their application are described in [51,52] (in this same journal issue).

### Prototype Signal Processing and Pattern Recognition Results

**Figure 3:**
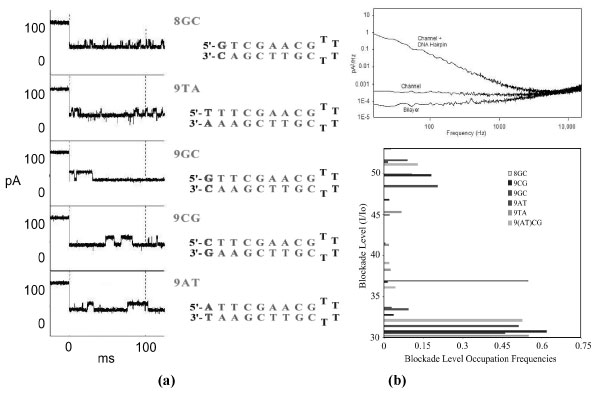
The channel current blockade signals observed in the prototype study (from [9]). Panel (a) shows the five DNA hairpins, with sample blockades, that were used to test the sensitivity of the nanopore device. Panel (b), top, shows the power spectral density for signals obtained from the different nine base pair DNA hairpins, as well as the open channel. Panel (b), bottom, shows the dominant blockades, and their frequencies, for the different hairpin molecules in panel (a).

**Figure 4:**
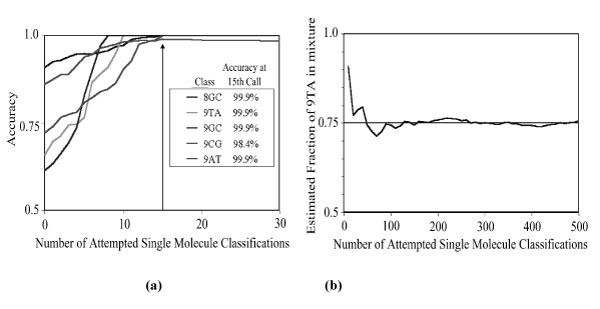
Panel (a) shows the single-species prediction accuracy as the number of signal classification attempts increases (allowing increase in the rejection threshold). Panel (b) shows the prediction accuracy on 3:1 mixture of 9TA to 9GC DNA hairpins (from [9]).

Five DNA hairpins were used in a prototype study to explore the sensitivity of the detector, as well as to probe the pore geometry [9]. The DNA hairpins studied (see Figure 3a) differed only in their terminal base pairs. Classification accuracy was 99.6% on average for the five DNA hairpins (see Figure 4a). The classification result for a mixture solution of 9TA and 9GC hairpin species (Figure 4b) is shown as the number of single molecule samplings is increased. The mixture was in a 3:1 ratio of 9TA:9GC, consistent with the 75% asymptote. Less than 1% error on majority population size was obtained by the hundredth observation (or, in approximately 100 seconds).

### Understanding the 9 base-pair DNA hairpin blockade mechanism

**Figure 5:**
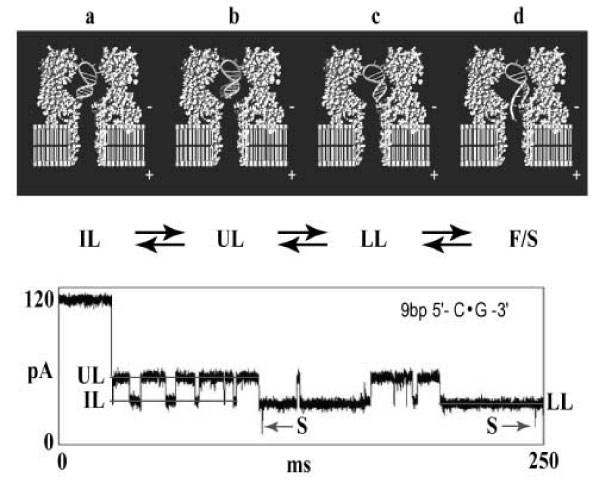
Shows the nine base pair DNA hairpin blockade mechanism (from [11]). When a 9 bp DNA hairpin initially enters the pore, the loop is perched in the vestibule mouth and the stem terminus binds to amino acid residues near the limiting aperture. This results in the IL conductance level. When the terminal base pair desorbs from the pore wall, the stem and loop may realign, resulting in a substantial current increase to UL. Interconversion between the IL and UL states may occur numerous times, or UL may convert to the LL state. The LL state corresponds to binding of the stem terminus to amino acids near the limiting aperture but in a different manner from IL. From the LL bound state, the duplex terminus may fray with rapid extension and capture of one strand in the pore constriction (denoted F/S).

Residual channel current decreases as blockading DNA hairpins increase their stem length from 3 to 8 base-pairs. For DNA hairpins with stems shorter than 8 base-pairs, multiple states were not clearly discernible, presumably because the hairpins were too short to bind to the channel favorably or interact with the current/force constriction near the limiting aperture. For 9 base-pair hairpins a clear 1/f noise (flicker noise) is discernible (Figure 3b, top) – a preliminary indication of the single-molecule binding kinetics described in [11] (and summarized in Figure 5).

**Figure 6:**
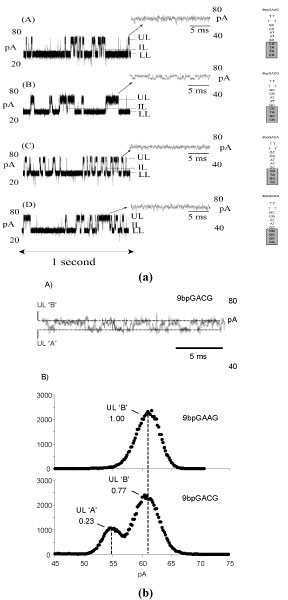
Preliminary results add credence to the hypothesis that the UL state has an unbound terminus, and that while in that state conformational switching may be observable (from [8]). For the molecules and blockades shown **(a) **above, three are found by NMR to have one dominant ground state (no switching), while one of the molecules is found by NMR to have two dominant low energy states (switching). This corresponds exactly with what has been observed in terms of the existence of fine structure (toggling) in the UL blockade state, as shown in **(b)**.

A critical understanding derived from the 9-base-pair DNA hairpin analysis is that if the UL blockade state is unbound at its terminus there is the possibility that conformational kinetics might be observable at the pore-captured polymer end. This motivated examination of a set of dsDNA termini that had already been examined using NMR. Results (Figure 6a &6b) show agreement with NMR via number of low energy conformational states observed [8].

### Nanopore Detector Augmentation for sticking

**Figure 7:**
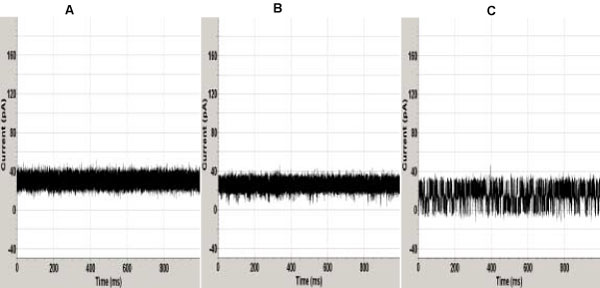
(A) 9GC DNA hairpin with 20 bp stem. (B) 9GC 20 bp stem with magnetic bead attached. (C) 9GC 20 bp stem with magnetic bead attached and driven by a laser beam chopped at 1 Hz. (From [7].)

A practical limitation of the nanopore device is that molecules to be detected, or specially designed blockade gauges (for indirect attachment) can blockade the channel and get "stuck" in one blockade state. One way of seeing how this might occur is to consider DNA hairpins with stems having 10, and more, base-pairs. As the hairpins are extended from 9 base-pairs, the clearly discernible toggle signal slows, and resides longer in the lower level (unpublished, originally observed by Dr. Vercoutere in the work leading to [12] but not presented there). Further work on hairpins with 10–12 base-pair stem lengths, by Dr. Vercoutere and other researchers collaborating on the foundational work in [12], is nearing completion (private communication), so this will not be discussed further here. When 14, or more, base-pair stems are attempted the resulting molecular blockades appear stuck in their lower level (at the time-scales of the experiment). The trend on blockade signals going from 14–20 base-pairs was not elaborated on in the analysis of the 20 base-pair dsDNA molecule that was the studied in [7], however, since that paper focused on how to recover highly structured blockade information from a minimally informative "stuck-state" situation. So there is a lot of interesting work yet to be done in this area. What was accomplished in [7] was to observe channel blockades due to a 20 base-pair DNA hairpin designed to have the same 9 base-pair terminus as the "sensitive" DNA gauge used in [8-12]. As expected, the 20 base-pair dsDNA molecule no longer modulates the channel flow, being stuck in just one blockade state. It is shown in [7], however, that the molecule can be re-excited to its telegraph signaling via a bead or some other form of attachment which is periodically tugged by a laser beam. In this way, the larger DNA hairpin has it's blockade signal "re-awakened" to reveal binding kinetic information at its channel-captured end (see Figure 7). This offers the prospect that simple excitations to the channel environment may enable having an analyte in a highly sensitive interaction (and detection) regime vis-à-vis the channel detector.

### Nanopore Detector Biosensing Augmentation using auxiliary molecules

The literature relating to nanopore biosensing all rely on mechanisms that involve a nanometer-scale channel and blockades of that channel. Some of the documents describe modifications to the channel geometry and its binding properties (thus sensing properties) by introduction of auxiliary molecules. The auxiliary molecules that modify the channel sensing environment can be covalently bound or non-covalently bound to the channel (cyclodextrins non-covalently bound [43] and short ssDNA oligomers covalently bound [53], for example). The design of these modifications is usually focused on providing alternative binding sites. In the case of auxiliary molecules covalently bound to the channel, the sensitive on-off binding kinetics between the molecule and the channel is eliminated entirely. In the case of the non-covalently bound (such as the cyclodextrins), the information pertaining to the on-off binding of the auxiliary molecule is not pursued in its own right. Instead, lengthy phases of fixed cyclodextrin blockade conformation are sought. In essence, the channel blockade states associated with analyte detection, or non-detection, can all be described in previous approaches as static blockades of the channel current. Typically, the binding or translocation of an analyte to the channel, or to the channel/auxiliary-molecule complex, is associated with a fixed reduction in the observed channel current. Little attention has been paid to the role of the auxiliary molecules vis-a-vis their on-off binding with the channel itself.

### Detection of short term binding and stationary phase

There are important distinctions in how a nanopore detector can function: direct vs. indirect measurement of static, stationary, dynamic (possibly modulated), or non-stationary channel blockades.

A nanopore-based detector can *directly *measure molecular characteristics in terms of the blockade properties of individual molecules – this is possible due to the kinetic information that is embedded in the blockade measurements, where the adsorption-desorption history of the molecule to the surrounding channel, and the configurational changes in the molecule itself, imprint on the ionic flow through the channel [7-12]. (Note: the hypothesis that the current blockade patterns are caused by adsorption-desorption, and conformational flexing, is not conclusively proven, although preliminary work on mechanism [11] and the success of the experimental approaches [7-12] add growing credence to this hypothesis.) This offers prospects for DNA sequencing and single nucleotide polymorphism (SNP) analysis. Nanopore methods may even have potential for *single-molecule *sequencing at some point in the future. Sequencing and SNP analysis, however, represent a small fraction of the immense potential of such a device. This is because a nanopore-based detector can also measure molecular characteristics *indirectly*, reporting on important binding kinetics in particular.

The nanopore-based detector works *indirectly *if it uses a reporter molecule that binds to certain molecules, with subsequent distinctive blockade by the bound-molecule complex. One example of this, with the established DNA experimental protocols, is exploration of DNA annealing or transcription factor binding sites via the different dsDNA blockade signals that occur with and without DNA annealing or DNA-binding of a hypothesized transcription factor (this is described further in the model DNA/DNA system and model DNA/Protein system sub-sections). Similarly, a channel-captured dsDNA "gauge" that is already bound to an antibody could provide a similar blockade shift upon antigen binding to its exposed antibody. The latter description provides the general mechanism for directly observing the *single molecule *antigen-binding affinities of any antibody.

### Nanopore observation of Conformational Kinetics on Model Biomolecules

**Figure 8:**
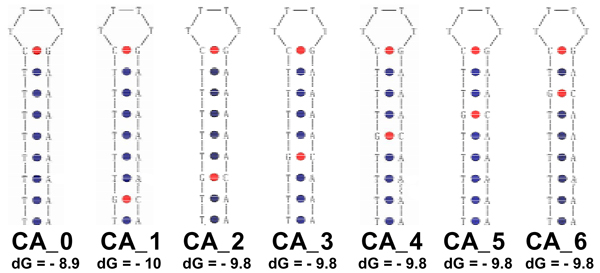
In preliminary nanopore studies the wild-type 3' end sequence (-C-A-T-G-3') was found to be similar to (-C-A-A-A-3'), which motivated the present study of a group of DNA hairpins consisting of all adenosines on the 3' side of the molecule, except for one cytosine-adenosine step. Contrary to the differences (seemingly) indicated by nature, the calculated ΔG° of hairpin formation (using MFold [54]) is the same for the set of molecules described, with one CA step (the CA set).

**Figure 9:**
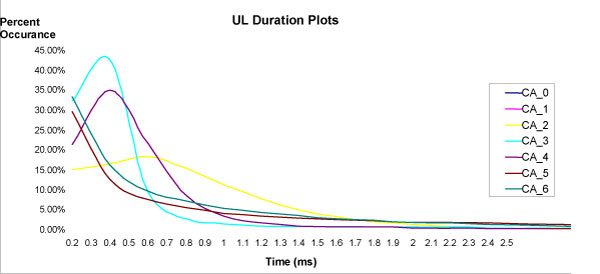
UL, the unbound terminus state, has shortest life for CA_3, i.e., CA_3 has strongest interaction with channel (and surroundings), neighboring variants (CA_2, CA_4) share this property to a lesser extent, and molecules with GC pairs more than 1 base-pair distant group very closely, the one molecule with no extra GC also separates with its own characteristic curve. This result is consistent with the increased reactivity of CA_3 to initiate complex formation [1], with weaker variants in CA_2 and CA_4, exactly as found experimentally [1-7].

Two conformational kinetic studies have been done, one on DNA hairpins with HIV-like termini, the other on antibodies. The work on antibody conformations is not complete, so is not described here, (but preliminary results on the antibody-antigen binding experiments *have *been obtained, and are described in that section). The objective of the DNA HIV-hairpin conformational study was to systematically test how DNA dinucleotide flexibility (and reactivity) could be discerned using channel current blockade information (see [26], in this same journal issue, for a complete description of the results pertaining to this study). The structural and physical properties of DNA depend upon nucleotide sequence, as is manifest in differences in three dimensional structure and anisotropic flexibility. Despite the multitude of crystallographic studies conducted on DNA, however, it is still difficult to translate the sequence-directed curvature information obtained through these tools to actual systems found in solution. Information on the DNA molecules' variation in structure and flexibility is important to understanding the dynamically enhanced DNA complex formations that are found with strong affinities to other, specific, DNA and protein molecules. An important example of this is the HIV attack on cells: one of the most critical stages in HIV's attack is the enzyme mediated insertion of viral into human DNA, which is influenced by the dynamic-coupling induced high flexibility of a CA/TG dinucleotide positioned precisely two base-pairs from the blunt terminus of the duplex viral DNA. This flexibility appears to be critical to allowing the HIV integrase to perform its DNA modifications. The CA/TG dinucleotide presence is a universal characteristic of retroviral genomes [27,28]. The behavior of the DNA hairpins containing the CA/TG dinucleotide at different positions relative to their blunt-end termini, is studied here using a nanopore detector. The nanopore detector feature extraction makes use of HMM-based feature extraction and SVM-based classification/clustering of "like" molecular kinetics. We hypothesize that the DNA hairpin with CA/TG dinucleotide, positioned two base-pairs from the blunt terminus, will have "outlier" channel current statistics qualitatively differentiable from the other DNA hairpin variants (the hairpins studied are shown in Figure 8). This is found to be the case (Figure 9), where the UL state, corresponding to the unbound terminus state, has shortest life for hairpin labeled CA_3 (in Figure 8). Since the UL state is hypothesized to be unbound, the fact that it has the shortest lifetime on average is an indication of the associated molecule's propensity to be bound to the channel (binding site is unknown at this time, although the work in [11] suggests some likely binding sites on the channel). In other words, CA_3 has strongest interaction with channel (and surroundings), as hypothesized, and neighboring variants (CA_2, CA_4), that have GC pairs shifted one base-pair shifted closer and further from the terminus, share this property to a lesser extent. Note: the "CA" notation refers to a dinucleotide step along the backbone of the self-annealed ssDNA strand in the hairpin molecule, while the GC base-pair described is part of that strand annealing, with the 'G' base-paired to the 'C' referred to in the CA-step (Fig. 8 should make this clear, with the GC base-pairing marked by a red bond, the AT pairs denoted by blue). It can be seen in Figrue 9 that the molecules with GC pairs more than 1 base-pair distant group vary similarly, the one molecule with no extra GC also separates with its own characteristic curve. This result is precisely consistent (Figure 9) with the increased reactivity of CA_3, and with weaker variants in CA_2 and CA_4, exactly as found experimentally in [27,28] – where it is observed that when the position of the dinucleotide pair **CA/TG **is altered slightly by mutation or in a synthetic substrate, the site of 3'-end processing and DNA joining, albeit weakened, moves correspondingly.

## Results

Results for binding kinetics experiments are described. The latest conformation kinetics results are summarized at the end of the preceding section. Please see [26], in this same journal issue, for a complete description of the results pertaining to experiments on conformational kinetics.

### Nanopore observation of Binding Kinetics on Model Biomolecular Systems

In the experiments described in Figure 12, Figure 15, Figure 16, and Figure 18, the sensing moieties (antibodies and aptamers) are themselves producing a very sensitive, rapidly changing, blockade signal due to their interaction kinetics with the channel environment (thus bifunctional in binding and desirable interaction kinetics). In the case of the antibodies, this simplification avoids the binding of antibody to specially designed DNA hairpin gauges, etc., at least for the antibodies examined, as they can interact with the channel and provide the sensitive "toggling blockade" signal needed themselves (see Antibody-Antigen sub-section). In the case of the aptamers, their toggling was by design (see Aptamer sub-section). A preliminary study of three model systems was performed:

#### I. The model DNA/DNA system: ssDNA annealing

**Figure 10:**
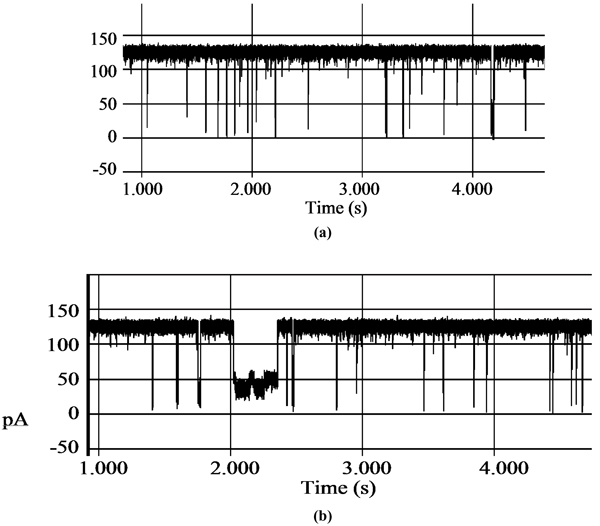
The preliminary aptamer experiments are based on the DNA molecule obtained from annealing ssDNA1: 5'-CAAGCTTGGTTTCGATAGGTA-3' with ssDNA2: 5'-ATCGTTTCCAAGCTTG-3'. For the pseudo-aptamer binding experiment a solution of annealed ssDNA1 and SSDNA2 molecules was exposed to ssDNA3: 5'-TACCT-3' (which anneals to the remaining AGGTA complement on ssDNA1). The target 5-base ssDNA is introduced subsequent to obtaining a toggler-type capture of the aptamer molecule (properly annealed). The aptamer experiment is referred to above as a pseudo-aptamer experiment due to its simplification to a DNA annealing detection. The short blockades in (a) result from ssDNA translocation by the un-annealed or partially annealed (mismatched) ssDNA components. The brief blockade in (b) is hypothesized to be due to capture with the overhang end entering first (and subsequent rapid melting of the annealed molecule – akin to that seem in the DNA hairpin experiments [12]).

**Figure 11:**
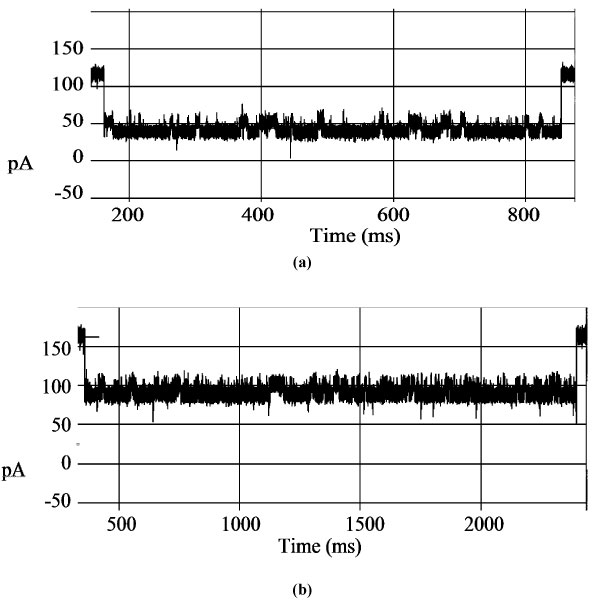
Panel (a) shows the sought-after toggler-type signal. Panel (b) Shows the modification to the toggler-type signal shortly after addition of 5-base ssDNA. Note: the blockade timescales are not the same, 0.7 seconds on (a) and 2.0 seconds for (b). The observed change is hypothesized to represent annealing by the complimentary 5-base ssDNA component, and thus detection of the 5-base ssDNA molecule. The "lengthy" toggler signal (a) was of briefer duration than desirable, by at least a magnitude, but modifications along the lines of the Y-aptamer geometries have recently yielded excellent results for characterizing binding properties (a work-in progress).

As a test case for DNA-DNA interaction studies with the nanopore detector, a "pseudo-aptamer" was examined (Figure 10 and Figure 11). Aptamers are nucleic acids selected for their ability to bind to molecules of interest and may provide the basis for a whole new class of medicines. If the aptamer is simply a DNA molecule with an overhang (a "sticky" end) then the segment of ssDNA that complements that overhang provides a known binding target with binding strength adjustable according to length of overhang, etc

#### II. The model DNA/Protein system: aptamers and sTF/TFBS

**Figure 12:**
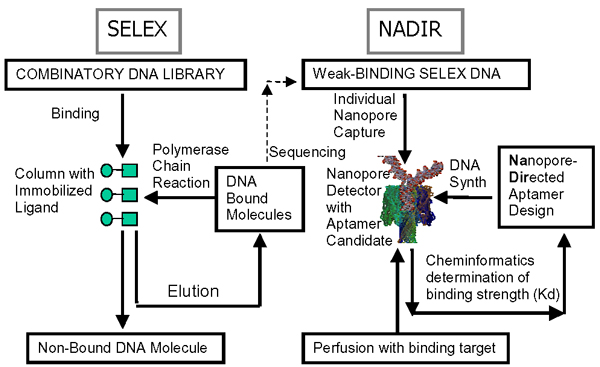
The determination of aptamers can be done (or initiated) via Systematic Evolution of Ligands by Exponential Enrichment (SELEX), as shown schematically on the left. What is proposed here is a linkage to a *na*nopore-detector *dir*ected (NADIR) search for aptamers that is based on bound-state lifetime measurements. NADIR complements and augments SELEX in usage: SELEX can be used to obtain a functional aptamer, and NADIR used for directed modifications (for stronger binding affinity, for example).

**Figure 13:**
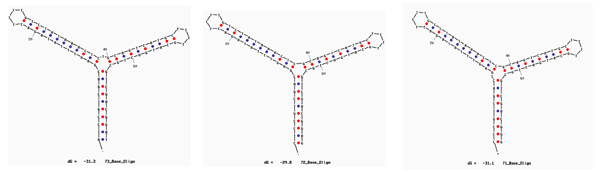
Shown are Y-shaped aptamers that have shown they have capture states with the desired blockaded toggling.

**Figure 14:**
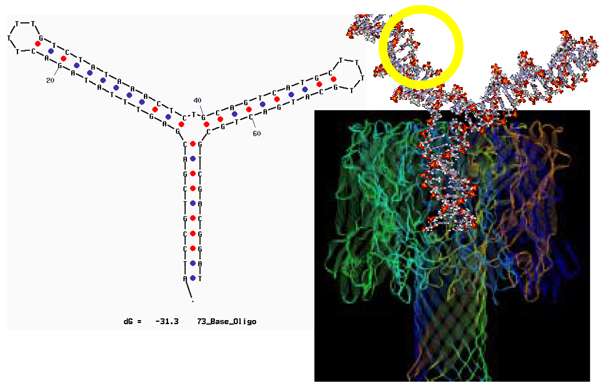
DNA Engineering: the goal is to design a bifunctional aptamer. Part of that bifunctionality hinges on a good reporter blockade (with stationary toggling). Such a blockade is partly brought about by having the Y-molecule caught at the mouth of the channel (such that the terminus of the dsDNA is over the limiting aperture). The other functionality is to bind to some target. The aptamer shown is designed to have a TATA box for TBP binding (the region indicated in the yellow circle).

**Figure 15:**
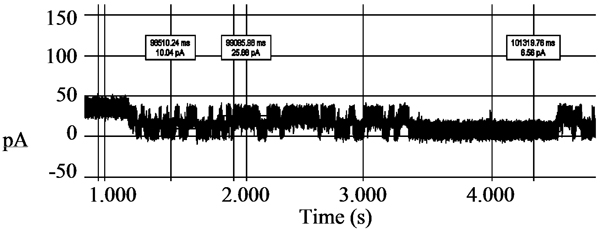
Multi-state terminus interaction with the channel limiting aperture -the desired telegraph or "toggle" signal.

A "reverse-engineered" TBP-binding aptamer (it has an exposed TATA box) is chosen. The first design problem, generic to all such NADIR aptamers (see Methods and Figure 12), is implementing the added functionality of toggle-blockade interactions with the nanopore. Some variants studied are shown in Figure 13. NADIR selection is constrained to the subset of aptamers that not only have desirable binding properties to their target, but that also have a common "base", partly consisting of a blunt 10 base-pair dsDNA end that provides a sensitive "toggle" signal upon capture by the electrophoretic forces at the nanopore's limiting aperture. This is accomplished by having the terminal base-pair perch over the limiting aperture due to a Y-branching in the DNA molecule being held at the channel's outer (cis) mouth (performing a similar role to the hairpin loop in prior, DNA hairpin, studies) [7-11]. As a test case for protein-DNA interaction studies, binding of TBP to an aptamer with a TATA binding site is studied with the nanopore detector, and preliminary results are shown (Figure 14 and Figure 15).

#### III. The model Protein/Protein system: antibody/antigen binding

**Figure 16:**
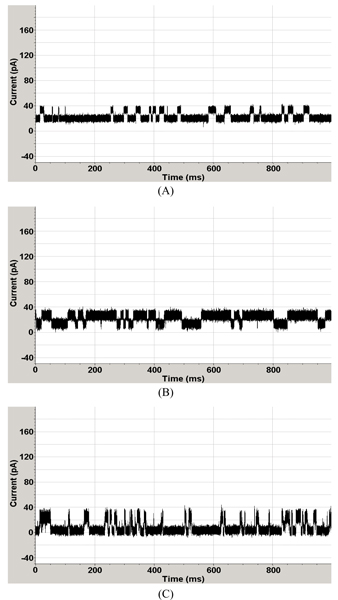
Single antibody, different blockade signals. (A) The lower-level (LL) blockade of this signal is not as deep as the others. (B) The LL blockade of this signal is about mid-way between those shown. (C) The LL blockade of this signal is the deepest, and difficult to eject with voltage reversal (requiring several attempts). The three signal classes represented by the above, as well as other, less common blockade signals (not shown), correspond to nanopore characterization of different epitopes of the antibody.

**Figure 17:**
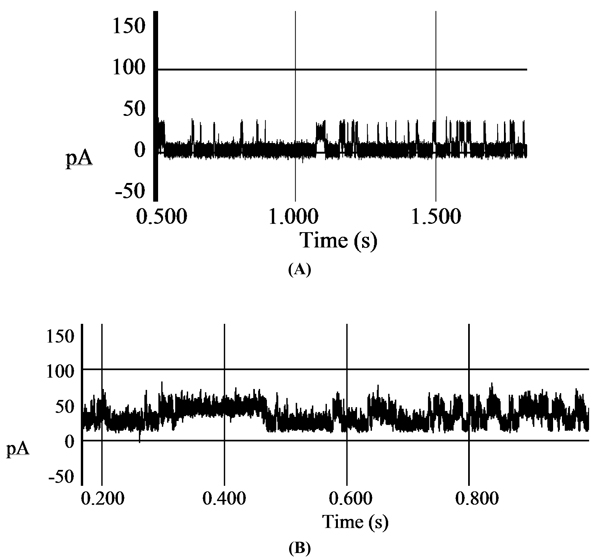
(A) Blockade due to T6 antibody. (B) T6 antibody blockade one minute after exposure to antigen.

Antibody conformational change, and antibody-antigen binding, are examined using a nanopore detector, and provide a test case for nanopore-based protein-protein interaction studies. Antibody blockade results with a variety of "toggle" signals, are shown in Figure 16. Blockades observed before and after introduction of target antigen are shown in Figure 17.

## Discussion

### Nanopore Detector Augmentation using bifunctional molecules

The improved detector sensitivity with toggling-type auxiliary molecules opens the door to a new, highly precise, means for examining the binding affinities between any two molecules (bifunctional or not), all while still in solution. In brief, an auxiliary molecule can be rigidly/covalently bound to the molecule of interest, and then exposed to a solution containing the other molecule of interest. The transitions between different stationary phases of blockade then be related to the bound/unbound configuration between the two molecules of interest to reveal their binding kinetics (and binding strength).

The bifunctional molecules that have been studied on the nanopore detector include antibodies and aptamers, and were chosen to demonstrate the specific utility of this device in drug candidate screening – antibodies that bind strongly to target antigen are good antibodies, same for aptamers in many situations. Sometime a weak-binding is desired, e.g., sometimes the drug is a toxin, so the strategy may be to deliver the toxin with a weak-binding agent such that the toxin may eventually be cleared.

In the larger context of the channel device itself, the advanced computational tools offer the possibility of analyzing/characterizing the device channels themselves (typically pore-forming toxins, such as from staph or anthrax). The advanced computational tools also offer a platform for examining the efficacy of certain channel-forming toxins (anthrax) as cellular syringes for delivery of specific molecules and drugs to the cellular cytosol (such as antigen delivery to evoke a CTL response).

### NADIR

In general, the determination of aptamers can be done (or initiated) via Systematic Evolution of Ligands by Exponential Enrichment (SELEX) [29]. What is proposed here is a "NAnopore-detector DIRected" (NADIR) search for aptamers that is based on bound-state lifetime measurements. NADIR complements and augments SELEX in usage (Figure 12): SELEX can be used to obtain a functional aptamer, and NADIR used for directed modifications (for stronger binding affinity, for example). In many respects, the DNA hairpins used in previous studies [7-11], and now used as controls, can be viewed as "dumb" aptamers in that they are nucleic acids with no binding properties. For the aptamer binding studies, where the choice of DNA aptamer is under the experimenters discretion, bi-functional aptamers are described that provide the desired "toggling" blockade signal with their captured ends (like the controls), while their uncaptured regions are designed to have binding moieties for various targets, i.e., annealing of a duplex DNA overhang to it compliment (see Figure 10 and Figure 11 for details).

### Deciphering the Transcriptome and Transcription-Factor based Drug Discovery

The examination of transcription factor binding to target transcription factor binding site (TF/TFBS interactions) affords the possibility to understand, quantitatively, much of the Transcriptome. This same information, coupled with new interaction information upon introduction of synthetic TFs (possible medicines), provides a very powerful, directed, approach to drug discovery. Synthetic transcription factors (STFs) promise to offer a powerful new therapeutic against Cancer, AIDS, and genetic disease. STFs that can appropriately target (and release) their transcription factor binding sites on native genomic DNA provide a means to directly influence cellular mRNA production (to induce death or dormancy for Cancer and AIDs cells, or restore proper cellular function in the case of genetic disease). In synthetic TF drug discovery, however, an effective mechanism for screening amongst TF candidates would itself be highly valued, and such may be possible with novel observation and analysis methods developed to study single molecule interactions/blockades of a single nanometer-scale channel.

### A new window into understanding antibody function

The basic domain structure of antibody is a "Y-shaped" elongated globular domain consisting of beta-pleated sheets. Antigen binding occurs in the amino-terminal domain (the tips of the arms of the Y), while the effector functions are localized to the C-terminal domains (the center of the Y). Upon binding to antigen, a series of events are initiated by the interaction of the antibody carboxy-terminal region (the base of the Y) with serum proteins and cellular receptors. Biological effects resulting from the carboxy-terminal interactions include activation of the complement cascade, binding of immune complexes by carboxy-terminal receptors on various cells, and the induction of inflammation. This provides a new way to study the binding/conformational histories of individual antibodies. Many critical questions regarding antibody function are still unresolved, questions that can be approached in a new way with the nanopore detector. The different antibody binding strengths to target antigen, for example, can be ranked according to the observed lifetimes of their bound states. Questions of great interest include: are allosteric changes transmitted through the molecule upon antigen binding? Can effector function activation be observed and used to accelerate drug discovery efforts?

Discussion of other experimental feedback control efforts, and nanopore implementation issues, has been placed in Appendix A.

## Conclusion

The results show great promise for observing interaction in solution on three model systems for DNA/DNA, DNA/Protein, and Protein/Protein interactions. The model DNA/DNA system consists of ssDNA (as an overhang) annealing to its ssDNA. The model Protein/DNA (aptamer) system consists of a DNA molecule with a transcription factor binding site, a TATA box, and a transcription factor (TBP protein) that binds to that site. The model Protein/Protein system consists of an antibody and its target antigen.

Channel current based kinetic feature extraction not only appears to be practical, but the next key step in the study of individual reaction histories. In essence, binding strength (Kd) between molecules in solution, and conformational state transitions, can be determined via channel blockade observations corresponding to lifetimes on the different states. The ergodic hypothesis, that time averages can replace ensemble averages, can now be explored in this context as well. Nanopore detection promises to be a very precise method for evaluating binding strengths and observing single-molecule conformational changes.

## Methods

### Nanopore Experiments

Each experiment is conducted using one α-hemolysin channel inserted into a diphytanoyl-phosphatidylcholine/hexadecane bilayer across a 25-micron-diameter horizontal Teflon aperture, as described previously [15] (Figure 1a). Seventy microliter chambers on either side of the bilayer contains 1.0 M KCl buffered at pH 8.0 (10 mM HEPES/KOH) except in the case of buffer experiments where the salt concentration, pH, or identity may be varied. Voltage is applied across the bilayer between Ag-AgCl electrodes. DNA control probes are added to the *cis *chamber at 10 or 20 μM final concentration. All experiments are maintained at room temperature (23 ± 0.1°C), using a Peltier device.

### Controlling Nanopore Noise Sources and Choice of Aperture

The accessible detector bandwidth is delimited by noise resulting from 1/f (flicker) noise, Johnson noise, Shot noise, and membrane capacitance noise. In Figure 3a, upper right, the current spectral density is shown for the typical bilayer, an open α-hemolysin channel, and a channel with DNA hairpin blockade. For 1.0 M KCl at 23 C, the α-hemolysin channel conducts 120 pA under an applied potential of 120 mV. The thermal noise contribution at the 1 GΩ channel resistance has an RMS noise current of 0.4 pA, consistent with Figure 3a. Shot noise is the result of current flow based on discrete charge transport. During nanopore operation with 120pA current (with 10 KHz bandwidth) there is, similarly, about 0.6 pA noise due to the discreteness of the charge flow. As with Johnson noise, the Shot noise spectrum is white, consistent with Figure 3a. The specific capacitance of lipid bilayers is approximately 0.8 μF/cm^2 ^(very large due to molecular dimensions), and the specific conductance is approximately 10^-6 ^Ω^-1^cm^-2^. In order for bilayer conductance to produce less RMS noise current than fundamental noise sources (under the conditions above), the leakage current must be a fraction of a pA. This problem is solved by reducing to less than a 500 μm^2 ^bilayer area, for which less than 0.6 pA leakage current results and for which total bilayer capacitance is at most 4pF. This indicates that a decrease in bilayer area by another magnitude is about as far as this type of noise reduction can go. Preliminary attempts to do this, however, lead to a very unpredictable toxin intercalation rate, among other difficulties.

### Control probe design

Since the five DNA hairpins studied in the prototype experiment have been carefully characterized, they are used in the antibody (and other) experiments as highly sensitive controls. The nine base-pair hairpin molecules examined in the prototype experiment share an eight base-pair hairpin core sequence, with addition of one of the four permutations of Watson-Crick base-pairs that may exist at the blunt end terminus, i.e., 5'-G•C-3', 5'-C•G-3', 5'-T•A-3', and 5'-A•T-3'. Denoted 9GC, 9CG, 9TA, and 9AT, respectively. The full sequence for the 9CG hairpin is 5'CTTCGAACGTTTTCGTTCGAAG3', where the base-pairing region is underlined. The eight base-pair DNA hairpin is identical to the core nine base-pair subsequence, except the terminal base-pair is 5'-G•C-3'. The prediction that each hairpin would adopt one base-paired structure was tested and confirmed using the DNA mfold server (, also see [54]), which is based in part on data from [42]. A standardized aliquot of antibody is used as the control for antibody experiments once the kinetics of antibody capture and antigen-binding events are established and shown to be highly reproducible.

### Data acquisition

Data is acquired and processed in two ways depending on the experimental objectives: (i) using commercial software from Axon Instruments (Redwood City, CA) to acquire data, where current was typically be filtered at 50 kHz bandwidth using an analog low pass Bessel filter and recorded at 20 μs intervals using an Axopatch 200 B amplifier (Axon Instruments, Foster City, CA) coupled to an Axon Digidata 1200 digitizer. Applied potential was 120 mV (*trans *side positive) unless otherwise noted. In some experiments, semi-automated analysis of transition level blockades, current, and duration were performed using Clampex (Axon Instruments, Foster City, CA). (ii) using LabView-based experimental automation. In this case, ionic current was also acquired using an Axopatch 200 B patch clamp amplifier (Axon Instruments, Foster City, CA), but it was then recorded using a NI-MIO-16E-4 National Instruments data acquisition card (National Instruments, Austin TX). In the LabView format, data was low-pass filtered by the amplifier unit at 50 kHz, and recorded at 20 μs intervals. In both fixed duty cycle (i.e., not feedback controlled) data acquisition approaches, the solution sampling protocol used periodic reversal of the applied potential to accomplish the capture and ejection of single biomolecules. The biomolecules captured consisted of antibodies and antigen, bound together or not, and in various orientations, and DNA control probes with stem-capture orientation and were added to the cis chamber typically in 20 μM concentrations. The time-domain finite state automaton [23] used in the prototype was used to perform the generic signal identification/acquisition for the first 100 ms of blockade signal (Acquisition Stage, Figure 1b). The effective duty cycle for acquiring 100 ms blockade measurements, when found to be sufficient for classification purposes, was adjusted to approximately one reading every 0.4 seconds by choice of analyte concentration. Further details on the voltage toggling protocol and the time-domain FSA are in Winters-Hilt et al., 2003 [9].

### Nanopore Detector Augmentation using bifunctional molecules

Nanopore Detector augmentation with bifunctional molecules brings a novel modification to the nanopore detection methodology. Now the idea is that the bifunctional auxiliary molecules produce a "toggling" blockade between several different levels (with two usually dominating). In other words, the resulting blockade signal for the auxiliary molecule by itself is no longer at approximately a fixed blockade level, but now consists of a telegraph-like blockade signal with stationary statistics. Upon binding of analyte to the auxiliary molecule (a binding site, or moiety, being its other functionality) the toggling channel blockade signal is greatly altered, to one with different transition timing and different blockade residence levels. Building on this as a binding affinity testing and biosensing platform requires sophisticated computational tools, such as Hidden Markov Models and Support Vector Machines, but offers at least a hundred-fold improvement to the sensitivity of the device. Given the noise in the system and the limited dynamic range for blockades of the open channel current, the device is greatly restricted if not endowed with the sensitive timing information. It has even been found that minor environmental alterations to temperature, pH, etc., results in the toggle signal produced by "toggling" type auxiliary molecule being modified significantly – in essence the channel with toggling-type auxiliary molecules can provide sensitive biosensing on the solution environment itself.

### Channel Current Signal Analysis & Pattern Recognition

**Figure 18:**
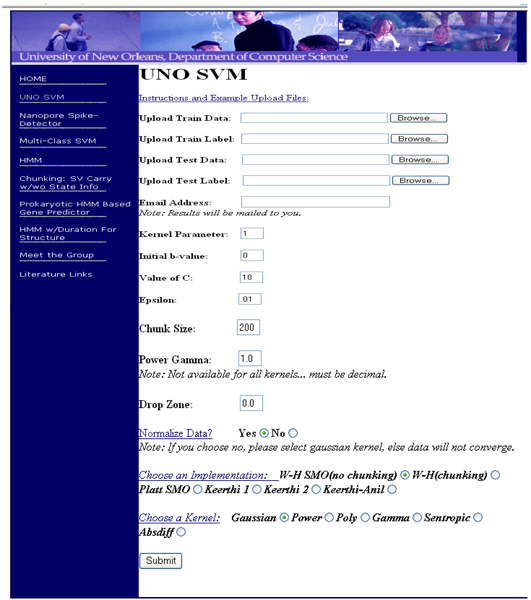
Several channel current cheminformatics tools are available for use via web interfaces at . These tools include a variety of SVM interfaces for classification and clustering (binary and multiclass), and HMM tools for feature extraction and structure identification (with applications to both channel current cheminformatics and computational genomics).

Extraction of kinetic information begins with identification of the main blockade levels for the various blockade classes (off-line). This information is then used to scan through already labeled (classified) blockade data, with projection of the blockade levels onto the levels identified with EVA projection (see [26]). A time-domain FSA performs the above scan, and uses the information obtained to tabulate the lifetimes of the various blockade levels. Once the lifetimes of the various levels are obtained, information about a variety of kinetic properties is accessible. If the experiment is repeated over a range of temperatures, a full set of kinetic data is obtained (including "spike" feature density analysis). This data may be used to calculate k_on _and k_off _rates for binding events, as well as indirectly calculate forces by means of the van't Hoff Arrhenius equation. Several channel current cheminformatics tools are available for use via web interfaces at  (see Figure 18). Further details on the Methods can be found in [26] in this same journal issue, and in [8,12].

## Appendix – Nanopore Implementation Issues

### Nanopore bio-sensor single-signal saturation

A limitation in the utility of the nanopore/antibody antigen-binding tester (similarly for a nanopore/aptamer tester) is that once antigen is bound by a channel-captured antibody it is very difficult to effect the release of that antigen. This is a complicating issue in acquiring a large sample of antibody-antigen binding observations. Attempts to "shake-off" the antigen by cycling to higher temperatures only serves to lead to added channel formation events – even after extensive perfusion of solution toxin upon the first channel formation. (This might result from higher mobility in the bilayer, at higher temperatures, that allows resident toxin monomers to coalesce on the timescale of the experiment that would otherwise not do so.) A buffer-based solution to this problem is already known from purifying antibodies through a column containing antigen, where the release of antibodies bound in the column is effected by perfusion with 1.0 M MgCl_2_. This presents the possibility of weakening the antibody-antigen binding by some choice of buffer in order to obtain large sample sets of binding events. The limitation of this is that the parameters will have likely deviated substantially from the physiological norm. Alternatively, a balanced stoichiometric ratio of antibody to antigen could be rapidly sampled, with lengthy sampling acquisitions only on antibody captures that occur without bound antibody and that then wait to observe antigen binding (this is the focus of an effort to link LabWindows experimental feedback control with the "in-house" channel current pattern recognition software, e.g., to know whether to take a lengthy sample, or eject the captured molecule and resample).

### Detection of brief state events – Automation and DNA Sequencing

In summary, when a biopolymer is present in an alpha-hemolysin channel it can produce a highly structured ionic current blockade pattern, where the lifetimes at various sub-blockade levels reveal information about the kinetics of the biopolymer (resulting from interactions with the channel, other molecules, or from conformational changes). LabVIEW/LabWindows automation has recently been integrated with the "in-house" Channel Current Cheminformatics (CCC) software, where data acquired with LabVIEW/LabWindows is passed to the CCC software on a streaming real time basis for analysis and classification. The classification results are then returned to the LabVIEW/LabWindows automation software for experimental feedback control. To accomplish this, data is "lifted" from the LabWindows environment, via a TCP/IP bridge, to a collection of Linux clients with the in-house CCC code. Classification results, in turn, are sent back, via TCP, to the LabWindows experimental controller. The first goal of this effort will be to provide feedback such that molecules are held until identified. Once identified (sometimes very quickly, in 100 ms), the molecule can be ejected or sampled more extensively, depending on the objectives of the experiment. This will increase the data-gathering efficiency of the current detector at least ten-fold.

### Nanopore Detector Stability

*Re-establishing the α-hemolysin channel on a day-to-day basis presents a major complication to the pattern recognition task*. SVM classification in such circumstances faces weaker training convergence and poorer signal calling. For the blockade signals considered, two stabilization approaches have been developed. (i) a passive stabilization approach that optimizes the kernels for high rejection. And (ii) active, computationally-based, stabilization methods that track control signature samples that are intermixed with the target analyte signal (see description of DNA hairpin controls in the Methods).

### Buffer/bilayer complications when working with protein analytes – use of S-layer mimicry

Complications of protein studies with the current nanopore device, in general, include the problem of the mismatch of a protein's isoelectric point with the desired electrophoretic response, and the problem of protein intercalation into the lipid bilayer. Addition of S-layer mimic component to the buffer, or directly into the bilayer via a preparatory coat step (used to introduce cholesterol, for example), may help to guard against intercalation, while other buffer modifications may serve to permit a more desirable electrophoretic response.

### Bandwidth limitations

Nanopore-based detection is limited by the kinetic time-scale of the molecular blockade states, where the molecular blockade states typically correspond to binding and dissociation (analyte-channel binding, or antibody-antigen binding, for example), or due to internal conformational flexing. It is hypothesized that it is possible to probe higher frequency realms than those directly accessible at the operational bandwidth of the channel current based device, or due to the time-scale of the particular analyte interaction kinetics, by modulated excitations. This can be accomplished by chemically linking the analyte or channel to an "excitable object", such as a magnetic bead, excited by laser pulsations, for example. In one configuration, the excitable object can be chemically linked to the analyte molecule to modulate its blockade current by modulating the molecule during its blockade. In another configuration, the excitable object is chemically linked to the channel, to provide a means to modulate the passage of ions through that channel. Studies involving the first, analyte modulated, configuration (Figure 7), indicate that this approach can be successfully employed to keep the end of a long strand of duplex DNA from permanently residing in a single blockade state. Similar study of magnetic beads linked to antigen may be used in the nanopore/antibody experiments if similar single blockade level, "stuck", states occur with the captured antibody (at physiological conditions, for example).

Examples of excitable objects include microscopic beads (magnetic and non-magnetic), fluorescent dyes, etc. Bead attachments can couple in excitations passively from background thermal (Brownian) motions, or actively by laser pulsing and laser-tweezer manipulation. Dye attachments can couple excitations via laser or light (UV) excitations to the targeted dye molecule. Large, classical, objects, such as microscopic beads, provide a method to couple periodic modulations into the single-molecule system. The direct coupling of such modulations, at the channel itself, avoids the low Reynolds number limitations of the nanometer-scale flow environment. For rigid coupling on short biopolymers, the overall rigidity of the system also circumvents limitations due to the low Reynolds number flow environment. Similar consideration also come into play for the dye attachments, except now the excitable object is typically small, in the sense that it is usually the size of a single (dye) molecule attachment. Excitable objects such as dyes must contend with quantum statistical effects (at the single-molecule level), so their application may require time averaging or ensemble averaging (where the ensemble case involves multiple channels that are observed simultaneously). In both of the experimental configurations, a multi-channel platform may be used to obtain rapid ensemble information. In all cases the modulatory injection of excitations may be in the form of a stochastic source (such as thermal background noise), a directed periodic source (laser pulsing, piezoelectric vibrational modulation, etc.), or a chirp (single laser pulse or sound impulse, etc.). If the modulatory injection coincides with a high frequency resonant state of the system, informative low frequency excitations may result, i.e., excitations that can be monitored in the usable bandwidth of the channel detector. Increasing the effective bandwidth of the nanopore device greatly enhances its utility in almost every application, particularly those, such as DNA sequencing, where the speed with which blockade classifications can be made is directly limited by bandwidth restrictions.

### Biological versus solid-state channels

The methods/experiments described here for biological channels are also applicable on solid-state platforms in a variety of incarnations – the underlying method remains the same: low-noise nanometer-scale ion channel measurements, together with a highly adaptable set of signal analysis and pattern recognition methods. Going forward, solid-state channels will allow greater control of components of the device physics, so will play a prominent role. The biological channels, on the other hand, are what are working right now, and they provide a plethora of interesting research milestones with the information gained about the biological systems themselves.

A complication with solid-state nanoscopic channels is that the technology for preparing them in solid-state media is still in its infancy [35]. Preparing a single channel in a silicon nitride, for example, is fraught with difficulties in maintaining a stable channel – partly because it is found that atomic flow occurs at the surface of the substrate with a subsequent opening or closing instability (depending on the initial size of substrate channel) [35]. Certain protein channels, on the other hand, are known to be very stable, and benefit from the fact that nature has designed them to self-assemble. Their biological origin has also led to their robust properties of heat dissipation, etc., upon polymer translocation, something that poses added complication in the solid-state setting. For these reasons, advances to date in nanoscopic-channel based detection have primarily involved single nanometer-scale protein channels ("nanopores") intercalated in lipid bilayer substrates.

A great deal of medical science stands to benefit from protein-channel-based experimentation, via both improved understanding of specific and general membrane constituents that contribute to pore formation, and via a new, nanopore-based, cheminformatics characterization of the many currently known protein channels. Areas of research accessible to nanopore-based cheminformatics include: ion channel and pore-forming toxin characterization; assays of molecules that confer beneficial modification to channel function; and understanding the pore-forming mechanism. In general, agents that stimulate membrane damage include not only bacterial pore-forming toxins (PFTs), but some enveloped viruses, animal and plant toxins, and man-made molecules and environmental pollutants [30]. PFTs, however, are one of the most potent and versatile weapons wielded by invading microbes [31]. A number of medically relevant pathogens are known to produce pore-forming proteins [32,33]. In many cases, PFTs are significant virulence agents: markedly reduced virulence is observed in mutant bacterial strains that have lost their ability to produce their PFT. Antibodies against the alpha-hemolysin PFT, for example, have been shown to protect against various *S. aureus *infections, while immunization against the PFT of the human pathogen *Aeromonas hydrophilia *leads to protection [34]. (Note, although PFTs directly have detrimental impact by destroying tissue cells and first-line immune cell defenses, their most deleterious impact is often less obvious, due to a wide spectrum of short- and long-range secondary reactions to the intracellular signaling confusion resulting from the lysed cells.)

### Membrane Environment and Alternative Channels for use in Biosensing and Screening

α-hemolysin is a membrane protein that forms an aqueous transmembrane pore. In order for such a pore to form in a lipid bilayer it is necessary for lipid molecules to be laterally displaced. For microbial pore-forming toxins, the energy used to drive the pore-formation process is thought to be solely provided by conformational changes in the toxin molecules themselves (i.e., the process is ATP-independent). For alpha-hemolysin, the energy needed for the pore-formation is shown to be due to the oligomerization of toxin monomers to form the channel heptamer complex [36]. Although pore-forming toxins, and membrane-permeabilizing molecules in general, have incredibly diverse sequence and structure, they all share in the same mechanism. They either directly intercalate into target membranes, or bind to particular target molecules in the membranes, and do so from a solution soluble monomeric form. They then assemble into multimeric, membrane-spanning pores. Attributes of the membrane, other than specific binding molecules, are often critical to this process, such as cholesterol-rich microdomains or lipid rafts. The role of cholesterol as a specific binding agent for individual formation events is well documented, and is required for pore-formation by many toxins (in natural setting, otherwise, channel formation can be activated by introduction of solvent, e.g., n-decane), including the Anthrax pore-forming toxin [37-39]. But cholesterol-rich microdomains also play a role in channel formation in a non-specific manner. This is due to the microdomains acting as concentration platforms that can aid in the assembly of proto-channel multimers as is described for the aerolysin heptamer in [34]. Once a proto-channel multimer has formed, such microdomains can also aid in the last step of transmembrane channel formation. This is due to the junctions between cholesterol-sphingolipid-rich domains and fluid-phase phosphoglyceride domains having locally favorable (weakened) bilayer characteristics that favor membrane penetration [40]. The opposite effect is also known to be medically relevant: unknown membrane constituents, or the lack thereof, can block an alpha-hemolysin heptamer complex from inserting a transmembrane functional domain. This is found to be the case for human granulocytes [41], where the agent preventing channel formation is unknown.

Cholesterol not only factors into protein-channel based detection as a specific and general pore-formation co-agent, but also, paradoxically, as a membrane strengthening agent in that it allows for greater vibrational shock resistance in the bilayer. For this reason, even though cholesterol is not required for α-hemolysin channel formation in bilayers, it is still important in nanopore experiments purely as a bilayer-strengthening agent, which also serves to reduce the (bandwidth limiting) membrane noise contribution in the nanopore detector by approximately 35%.

### Multi-channel biosensing

The single-channel biosensing methods used here can be generalized to where many channels are present, where each channel offers parallel conductance paths for the ionic current, and where each channel is augmented with antibody (or aptamer) to establish a background collection of channel/antibody signals that is modifiable in the presence of antigen. Multi-channel methods offer similar capabilities to surface plasmon resonance approaches to characterizing binding affinity. Multiple antibody (aptamer) species can be present in this multi-channel operation. Anything that can evoke an antibody response (or SELEX cultivation, for aptamers) can be taken as the antigen or collection of antigens for which the bio-sensing is designed.

One application of multi-channel biosensing is in the field of bio-defense – a fractionated bio-terror organism, such as fractionated anthrax spores (i.e., pieces of protein, etc.), may be injected into a mouse to evoke a broad-spectrum antibody response. A collection of nanopore/antibody channel currents could then be prepared with those antibodies for detection of fractionated anthrax spores obtained via an air-concentrator with the same protein fractioning processes. Introduction of the fractionated air-concentrate when spores are present to the nanopore/antibody detector is hypothesized to evoke a discernible indication of the presence of that microorganism.

### Immunological Screening for Pore-Inhibiting Agents using CCC

A nanopore cheinformatics detection platform provides a new means for identifying pore inhibiting agents. This has profound medical significance for identifying treatments against pore-forming toxins associated with disease, bacterial and viral infection, and chemical exposure. In this experimental effort a pore-forming toxin, or membrane-permeabilizing agent, will be introduced into a membrane. In one experimental approach, the lifetime of a single toxin channel will be characterized prior to its disruption by the pore inhibiting agent being screened. The method also enables one to screen for agents that might prevent pore formation, by examination of the average time before channel formation under identical circumstances (pore forming toxin w/wo pore inhibiting agents).

A multiple toxin channel version of the experiment can be performed as well, with disruptions to their cumulative currents studied. Alternatively, toxin can be introduced and the timescale of multi-channel formation examined in the presence of (purported) pore inhibiting agent. The multi-pore experimental modes offer the possibility of binding affinity characterization on very fast time-scales, while the single-pore experiments provide *single*-molecule information about the action of the pore inhibiting agents. The single-channel experiments done initially can also be used to inform the design and statistical modeling of the multi-channel experiments. Pore-inhibiting agents that can be studied in this manner include antimicrobial peptides, the larger family of amphipathic molecules, antibodies, and aptamers.

Medicines and vaccines that provide resistance to pore-forming and membrane permeabilizing toxins can be rapidly assayed using methods like those just described. Pore-forming toxins are often virulence factors associated with bacterial and viral infections, or with toxic chemical exposure. The medicines obtained can either provide resistance to pore-forming toxin prior to their pore-formation or act as treatment after exposure to pore-forming toxins.

### Nanopore-based assays of cytosolic antigen delivery complexes

Nanopore-based detection may provide an effective method for identifying good cytosolic antigen delivery complexes for use in evoking cytotoxic T lymphocyte (CTL) responses in organisms challenged by cytosolic virulence factors. In all such screening efforts, pore-forming toxins are used that allow introduction of peptides and other molecules into the cytosol of their host cell. The experiments being designed entail establishing a single pore-forming toxin in a membrane followed by measurement of peptide (antigen) transmembrane transport via channel current measurements. The planned experiments make use of established procedures for attaching target antigen to the recognition sequence of virulence factors associated with the pore-forming toxin in the natural setting. Via this transmembrane transport mechanism, a number of antigen/recognition molecules will be assayed for effective use with the chosen pore-forming toxin.

Medicines and vaccines that provide resistance to cytosolic virulence factors can be rapidly assayed with this approach. Pore-forming toxins and viruses (e.g., HIV) are often associated with cytosolic virulence factors. Medicines and vaccines can provide treatment or resistance to such cytosolic virulence factors if an effective mechanism is devised for delivery of virulence-associated antigen to the cytosol of the host cell. Effective delivery of antigen to the cytosol is the first step in obtaining an effective agent for evoking a CTL response against virulence factors of the invading microorganism.

### Characterization under physiological buffer conditions

The standard buffer condition for the nanopore detector is 1.0 M KCl with a pH of 8.0. This buffer was found to be most conducive to channel formation and to channels that do not gate. At significantly lower pH the channel is known to gate [30], if it even forms in the first place, which complicates use of the nanopore detector at physiological conditions (pH 7.0, 100 mM NaCl). Since the pH of blood is usually in the range 7.35 to 7.45, and channel formation has been observed at 250 mM KCl, nanopore operation at the high pH and high salt end of the physiological range, relevant for antibody function in the bloodstream, may be possible with minimal alteration to the experimental parameters. Evaluation of antibody/antigen binding efficacy in a physiological buffer environment is particularly important if the nanopore/antibody detector is to be used for clinically relevant screening on the efficacy of antibodies to a given antigen.

If unable to alter buffer to physiological conditions, this will alter the on and off rates of antigen binding. But ELISA studies have shown that the antibodies still bind to the multivalent forms of the antigen in 1 M KCl, although binding is diminished, and it appears that one may be able to lower the KCl concentration to 0.25 M and still get pore formation. Thus while the validity of rate constants and other parameters of the interaction may be called into question, one may still be able to perform channel analyses, and other studies of antibody capture and alteration of channel blockade by antigen binding.

### Automated Feature Selection

Two new methods are being pursued for automated feature selection/feature compression. This is particularly important for handling the transition probabilities obtained by the HMM (if it has 100 states, it has 1002 transition probabilities). The main approach being developed is to use boosting (AdaBoost) over the individual emission and transition probabilities (which are used to provide a pool of weak, naïve Bayes, classifiers) to select the best features, then use those features when passing feature vectors to the SVM classifiers. A comparison case will be the HMM-template approach (avoided thus far, due to non-scalable issues, but will provide true challenge for the boosting approach – which doesn't have the scaling problem.).
